# The Acceptability and Usability of Digital Health Interventions for Adults With Depression, Anxiety, and Somatoform Disorders: Qualitative Systematic Review and Meta-Synthesis

**DOI:** 10.2196/16228

**Published:** 2020-07-06

**Authors:** Shireen Patel, Athfah Akhtar, Sam Malins, Nicola Wright, Emma Rowley, Emma Young, Stephanie Sampson, Richard Morriss

**Affiliations:** 1 Division of Psychiatry and Applied Psychology, School of Medicine University of Nottingham Nottingham United Kingdom; 2 School of Social Sciences Birmingham City University Birmingham United Kingdom; 3 Faculty of Medicine, School of Health Sciences University of Nottingham Nottingham United Kingdom; 4 School of Medicine University of Nottingham Nottingham United Kingdom; 5 Nottinghamshire Healthcare NHS Foundation Trust Nottingham United Kingdom; 6 NIHR MindTech MedTech Co-operative, University of Nottingham Nottingham United Kingdom

**Keywords:** digital health interventions, depression, anxiety, somatoform disorders, smartphone, mobile phone

## Abstract

**Background:**

The prevalence of mental health disorders continues to rise, with almost 4% of the world population having an anxiety disorder and almost 3.5% having depression in 2017. Despite the high prevalence, only one-third of people with depression or anxiety receive treatment. Over the last decade, the use of digital health interventions (DHIs) has risen rapidly as a means of accessing mental health care and continues to increase. Although there is evidence supporting the effectiveness of DHIs for the treatment of mental health conditions, little is known about what aspects are valued by users and how they might be improved.

**Objective:**

This systematic review aimed to identify, appraise, and synthesize the qualitative literature available on service users’ views and experiences regarding the acceptability and usability of DHIs for depression, anxiety, and somatoform disorders.

**Methods:**

A systematic search strategy was developed, and searches were run in 7 electronic databases. Qualitative and mixed methods studies published in English were included. A meta-synthesis was used to interpret and synthesize the findings from the included studies.

**Results:**

A total of 24 studies were included in the meta-synthesis, and 3 key themes emerged with descriptive subthemes. The 3 key themes were initial motivations and approaches to DHIs, personalization of treatment, and the value of receiving personal support in DHIs. The meta-synthesis suggests that participants’ initial beliefs about DHIs can have an important effect on their engagement with these types of interventions. Personal support was valued very highly as a major component of the success of DHIs. The main reason for this was the way it enabled individual personalization of care.

**Conclusions:**

Findings from the systematic review have implications for the design of future DHIs to improve uptake, retention, and outcomes in DHIs for depression, anxiety, and somatoform disorders. DHIs need to be personalized to the specific needs of the individual. Future research should explore whether the findings could be generalized to other health conditions.

## Introduction

### Background

The prevalence of mental health disorders continues to rise [1]. It is estimated that globally in 2017, 264 million people (3.4%) experienced depression, and 284 million (3.7%) of the population experienced an anxiety disorder. This included phobias, obsessive-compulsive disorder (OCD), posttraumatic stress disorder (PTSD), or generalized anxiety disorder (GAD) [2]. The worldwide prevalence of somatoform disorders or Medically Unexplained Symptoms in Primary Care is 25%-50% [3]. From an economic and social perspective, the costs of mental health disorders are high. The total costs of mental ill health are estimated at more than 4% of the gross domestic product, or over €600 (US $650.31 million), across the 28 EU countries [4]. More than two-third of individuals with depressive disorders also have anxiety symptoms, and 40%-70% simultaneously meet the criteria for at least one type of anxiety disorder [5,6]. Depression, anxiety, and somatoform disorders are often comorbid, and treatment can reduce symptomology in all 3 areas [7], implying that treatments could be effective across comorbid mental health conditions. Furthermore, where large-scale mental health interventions have been implemented, there is evidence of coaction—where a change in one disorder is achieved, it is more likely that change will be achieved with other health disorders [8].

Despite the high prevalence, only 33% of adults with depression and anxiety receive treatment in England [9], indicating that few people who need treatment receive it. The barriers to accessing psychological therapy include a shortage of therapists, long waiting times, and the stigma of accessing psychological treatment [10]. A recent authoritative review identified the use of digital health interventions (DHIs) as central to addressing population-level mental health. The absence of widely disseminated, accessible mental health interventions has also been identified as a key reason for the lack of improvement in mental health treatment [8]. DHIs have the potential to improve accessibility and meet the requirements expected from mental health services [10].

DHIs can be defined as interventions that provide information, therapy, and support (emotional, decisional, or behavioral) for physical or mental health disorders via a technological or digital platform (website, computer, mobile phone app, SMS text message, email, videoconferencing, wearable device, patient portals, or personal health records or virtual reality [VR]) [11,12]. The variety and functionality of digital health technologies continue to evolve. The forms of DHIs include computerized cognitive behavioral therapy (cCBT) but can also include telehealth or telemedicine utilizing telecommunications processes such as SMS text messaging, email, and videoconferencing for remote delivery of therapy. Recently, there has also been the emergence of *mHealth* or mobile health, which incorporates smartphone apps, remote monitoring, and wearables. DHIs can be standalone interventions, used as a stepped care model, provided in addition to face-to-face treatment or mediated by a health care professional such as a therapist [13,14].

Numerous systematic reviews have found that for people with depression and anxiety disorders, DHIs can be just as effective as face-to-face therapy [15,16]. However, dropout rates for completion of computerized psychological therapy are high [17,18]. Waller and Gilbody [17] conducted a systematic review of the quantitative and qualitative evidence identifying the barriers to the uptake of cCBT. They concluded that, a median of, 38% (range 4%-84%) of participants who were recruited started cCBT, and a median of only 56% (range 12%-100%) of starters completed it. They concluded that these low figures were influenced more by personal circumstances than technological aspects. Waller and Gilbody [17] acknowledged that more data was needed to explore why so few started or discontinued therapy. The inclusion of therapist support and guidance has been found to improve treatment completion rates and increase treatment effect sizes [19-21], implying that therapist-supported DHIs rather than self-guided DHIs may lead to more positive experiences for participants. The concept of blending face-to-face therapy with computer-delivered therapy or via videoconferencing has recently begun to emerge, which attempts to address some of the barriers to engaging with DHIs [22-24]. Furthermore, a recent article [25] identified that the top 10 priorities for DHIs in mental health include considering how DHIs could be combined with human support to improve its effectiveness.

Existing qualitative systematic reviews provide insight into the potential barriers and facilitators of recruiting and retaining participants in DHI research trials. O’Connor et al [26] investigated the factors affecting patient and public engagement and recruitment to DHIs. They identified that there was a need for greater awareness and understanding about DHIs to improve public understanding of DHIs [26]. They also recommended that DHIs need to incorporate some kind of social interaction to improve recruitment. DHIs also need to be tailored to individual needs. Knowles et al [27] explored user experience of digital technologies as a low-intensity intervention delivered with minimal or no professional support for depression and anxiety, concluding that personalization and tailoring content toward users’ needs improved the experience of computerized therapy. Furthermore, they recommended for future research to explore if these findings could be extrapolated to other delivery formats and treatment modalities.

Given the rise in prevalence of depression, anxiety, and somatoform disorders and advances in the development of DHIs, but low uptake and completion rates, it is important to conduct a systematic review exploring perceived barriers and facilitators to the acceptability and usability of a range of DHIs. This systematic review aimed to build on previous literature to explore the diverse nature of DHIs and whether potential facilitators and barriers are consistent across different types and formats of DHIs. We wanted to investigate whether there were specific issues relevant to the integration of support compared with self-guided DHIs and whether varying levels of support, intensity, and delivery formats influenced patient experience.

This is the first meta-synthesis to our knowledge that will look at the acceptability and usability of DHIs across different types and formats and across a range of depression and anxiety disorders. This systematic review includes all DHIs regardless of the delivery method (ie, text-based, automated, blended therapy). It compares very different experiences to consider whether therapies involving digital aspects share common issues and if consistent themes are found across different formats and modes of delivery. A systematic review of the qualitative literature may provide further explanation and synthesis of factors that may facilitate adherence and outcome of DHIs for comorbid mental health conditions. The key findings of this systematic review will inform the design of mental health digital interventions. This will support the development of digital mental health interventions tailored to service users’ needs and thus improve uptake, adherence, and experience, which will have a positive impact on service users and providers.

### Objectives of the Systematic Review

This systematic review aimed to understand the experiences of service users with regard to DHIs for depression, anxiety, and somatoform disorders. Specific objectives were to systematically identify, appraise, and meta-synthesize available qualitative literature that explored the following:

1. Service user’s perceptions of DHIs regarding their acceptability and usability.
2. Aspects of DHIs that are valued and work well and those that could be improved or altered.
3. Why service users chose to use, continue with, or stop using DHIs.

## Methods

The protocol for the systematic review was published on the International Prospective Register of Systematic Reviews (ID: CRD42018104016) before completion of the systematic review. The systematic review is reported in accordance with the Preferred Reporting Items for Systematic Reviews and Meta-Analyses (PRISMA) [28] statement checklist and Transparency in Reporting the Synthesis of Qualitative Research [29] guidelines (Multimedia Appendices 1 and 2).

### Search Strategy

A comprehensive search strategy was developed with an expert health information specialist (EY). A scoping search was conducted to identify key papers and associated search terms to inform the design of the search strategy. A systematic search was conducted for published papers that contained qualitative information about service user experiences of participating in a DHI trial for depression, anxiety, or somatoform disorders. A combination of free terms and controlled vocabulary terms was used to ensure that all relevant studies were identified. Search terms were split into 2 categories: DHIs and mental health conditions (depression, anxiety, and or somatoform disorders). Qualitative search filters identified by the InterTASC Information Specialists Sub-Group were also applied [30]. The search strategy was developed for MEDLINE (Multimedia Appendix 3) and then adapted and applied to PsycINFO, Cumulative Index for Nursing and Allied Health Literature (CINAHL), EMBASE, ISI Web of Science, Scopus, and the Cochrane Library. We searched the electronic databases for eligible studies from database inception to July 17, 2018. The search was conducted by EY. From the search results, papers that were published in the same study but in different publications were not removed to determine more closely if they reported different data aspects of the research. Search results were uploaded and stored using EndNote version X8.

### Inclusion and Exclusion Criteria

Textbox 1 describes the inclusion and exclusion criteria.

**Inclusion criteria and exclusion criteria.**

**Inclusion criteria**

- English language
- Community, primary, and secondary care
- Original qualitative studies, studies involving secondary analysis of qualitative data, or qualitative studies that were part of a mixed methods study (eg, the study also had a quantitative component, but the major component was qualitative, and a qualitative methodology was described). Studies should include a substantial amount of qualitative methods, including interviews, observations, and open-ended evaluation forms. Free textboxes on evaluation forms were included if there was richness in the data provided (ie, sufficient quotes to support the analysis)
- Papers should include some form of qualitative data analysis such as thematic or inductive analysis
- Papers reporting on participants who had experienced the use of digital health interventions (DHIs, also called *internet interventions* or *electronic health interventions*), where the DHI was primarily used to treat depression, anxiety, and/or somatoform disorders. This included interventions that provided information and support (emotional, decisional, and/or behavioral) via a technological or digital platform (website, computer, mobile phone app, SMS text message, email, videoconferencing, wearable device, patient portals or personal health records, or virtual reality)

**Exclusion criteria**

- Gray literature or literature not published in a peer reviewed journal
- Dissertation or theses
- Published abstracts or conference proceedings
- Any type of literature review, systematic review, and meta-synthesis
- Experiences of health care professionals or parents or carers
- Studies where the primary digital health interventions (DHIs) were telephone-based with no additional technological function (eg, telephone counseling or triaging service), internet-based health tools that were not defined as interventions (eg, internet health searching), or an implantable device that was remotely monitored
- Interventions to improve adherence to medication, improve assessment or diagnosis, or where digital interventions were not the major constituent of the intervention
- Peer-to-peer networks and DHIs of social support via the internet, use of social media, online support groups, or DHIs consisting of group therapy
- Data collected during the testing of the usability and design of DHIs
- Males and females aged <18 years; studies were included if ≥50% of the sample were aged ≥18 years

### Screening and Data Extraction

#### Data Screening

Duplicated studies were removed in EndNote, and the remaining articles were exported into a Microsoft Excel document for screening. Qualitative results from the same overall study that were split across different publications were not removed until the full-text screening. Papers were removed if they reported the same qualitative findings; otherwise, they were included. In addition, we carried out backward citation by hand searching the references of all included studies and key papers. Forward citation searching of the included studies was carried out in Scopus. Initial screening of titles and abstracts was conducted by 1 reviewer (SP), with a second reviewer (AA) screening a random 10% sample to confirm congruence. Interrater agreement for full-text screening was 99.4%, and disagreements were resolved through discussion.

#### Data Extraction

Data were extracted by SP from papers included in the systematic review using a data extraction form developed by SP and AA. The primary focus of data extraction was the identification of specific qualitative findings—reported themes and subthemes related to the phenomena of interest, which were subsequently synthesized as described below. All text from the papers labeled as results or findings were entered into a Microsoft Word document. Additionally, descriptive data, including details about DHIs, study aims, methods and analysis, country of research, and demographics of participants, were extracted. The form was initially piloted on 3 papers by both reviewers. AA completed data extraction for 30% of the articles to confirm congruence. Data extraction forms were compared to ensure data accuracy and comprehensiveness. Any disagreements were resolved through discussion until a consensus was reached.

### Quality Appraisal

Quality assessment of papers included in the meta-synthesis was undertaken by SP and AA using the Critical Appraisal Skills Programme (CASP) criteria [31]. Studies were not excluded on the basis of quality because we were more concerned about including papers that contained depth in data collection and analysis, which might provide valuable information regarding participant experience of DHIs. We included papers that collected data through semistructured interviews. We included papers that collected data through open response text if there was richness in the data provided.

### Meta-Synthesis

A meta-synthesis approach was used to organize and interpret the data. The findings of the included studies were synthesized using methods proposed by Noblit and Hare [32]. Papers were read and re-read by SP and AA, and first- and second-order constructs were extracted using a Microsoft Word template form. First-order constructs were defined as direct participant quotes reported in the papers. Second-order constructs were defined as the authors’ interpretations of participants’ quotes expressed as themes extracted from both the results and findings sections of papers. SP and AA independently sifted the second-order constructs, compiling new third-order constructs that summarized and encompassed the various themes across studies. Third-order constructs refer to synthesized constructs that emerge from the analysis of first- and second-order constructs. Constructs were reviewed to see how the themes were similar when compared across papers to make sense of the variability in participant experience of DHIs. A draft summary of the analytical themes was written by SP and AA and shared with the review team consisting of service providers and service user representatives. Themes were refined until consensus was reached.

## Results

### Summary of Search Results

The initial search yielded 9030 records. After duplicate records were removed, there were a total of 8936 records. Following title and abstract screening, 8836 articles were excluded, resulting in a total of 100 papers being reviewed in full; 1 paper that was not identified in our search but included in a key systematic review paper [27] was included [33], and 1 paper was identified and included through hand searching [34]. A total of 26 papers met the eligibility criteria and were included in the meta-synthesis. These were amalgamated into 24 studies because 2 of the included studies reported data in 2 papers. One study that was included was reported in a qualitative paper as part of a Health Technology Assessment report, the themes highlighted in the 2 papers differed, and both findings were included [35,36]. For another study that was reported in 2 papers, 1 paper specifically focused on motivation to persist with the DHI, while another paper focused on patient experience and the implementation of digitally delivered cognitive behavioral therapy (CBT) [37,38].

The majority of the studies were nested within a randomized controlled trial (RCT; n=15). All the included studies were deemed to be of sufficient quality to contribute to the meta-synthesis. All papers reported a clear statement of the aims of the research and were deemed to be valuable in their contribution to the themes. Question 6 of the CASP referring to the relationship between the researcher and participants was acknowledged in only 13 papers. Kuhn et al [39] contributed the least to the meta-synthesis because the paper provided minimal information regarding the data analysis method and how themes were derived (Multimedia Appendix 4).

**Figure 1:**
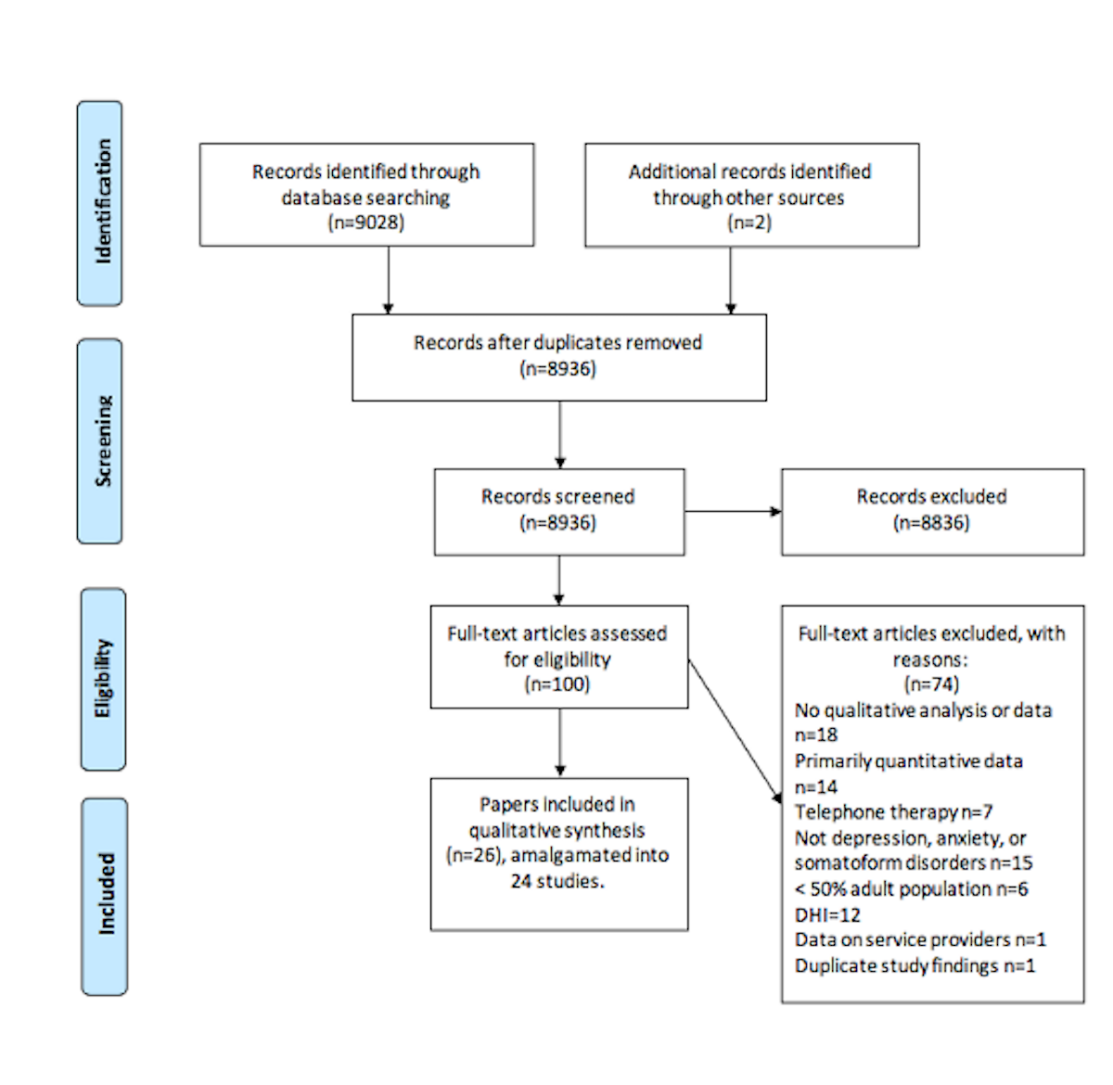
Flow diagram of study identification and selection adapted from Preferred Reporting Items for Systematic Review and Meta-Analyses (PRISMA).

The PRISMA diagram (Figure 1) illustrates the flow of study identification and selection. The main reasons for exclusion included no qualitative analysis or data, primarily quantitative data, primary focus not being depression, anxiety, or somatoform disorders, and lack of data on the experience of using a DHI. Multimedia Appendix 5 summarizes the included studies. The sample characteristics of the included studies are described in Multimedia Appendix 6.

The included studies were published between 2007 and 2018. The majority of the studies were carried out in European countries, primarily England and Sweden (n=11). A total of 13 studies looked at all types of depressive disorders, including major depression and dysthymic disorder, postpartum depression, and studies where depression was comorbid with cardiovascular disease and multiple sclerosis; 6 studies examined anxiety disorders, including panic disorder, PTSD, OCD, GAD, and postpartum anxiety; and 4 studies examined depression and/or anxiety. No qualitative studies have been conducted on the use of a DHI for the treatment of somatoform disorders. The majority of the participants were recruited from the community, and 1 study recruited participants from a multiple sclerosis outpatient clinic. Only 11 papers reported the ethnicity of participants. The participants in the studies were primarily of a white ethnic background and younger or middle-aged. The majority of the studies collected data via interviews, and 2 studies collected data via open-ended questionnaires. Of the total, 18 studies were purely qualitative studies, and 6 studies were mixed methods studies.

The studies varied in types and formats of DHIs. Of the 24 studies, the majority provided additional support via email, telephone calls, or SMS text messages (n=13); 6 studies included some form of face-to-face support, whereas 5 studies were purely self-guided DHIs with no support provided. In relation to platforms, the majority of the studies were DHIs accessible on desk-based computers (n=19). Only 1 study used a blended approach consisting of video-based therapy. Of the eligible studies, 2 studies consisted of smartphone apps, and 1 study consisted of web- and smartphone-based monitoring. Only 1 study included the use of a computer telephony system designed to monitor and support self-care. In terms of treatment approach, the majority of studies were based on CBT principles (n=19).

### Results of Meta-Synthesis

The meta-synthesis revealed 3 major themes and 9 subthemes related to the 3 objectives of the systematic review:

1. Service user perceptions of DHIs regarding their acceptability and usability.
2. Aspects of DHIs that are valued and work well and those that could be improved or altered.
3. Why service users chose to use, continue with, or stop using DHIs.
4. Selected supporting quotations from across the studies is provided in Multimedia Appendix 7 to illustrate this meta-synthesis. It shows first-, second-, and third-order constructs and synthesized subthemes with contrasting positive and negative participant experiences of DHIs.

The first theme, *initial motivations and approaches to DHIs*, had 2 subthemes:

1. Initial motivations: hope, accessibility, and cynicism.
2. Participant approaches to engaging with a DHI: active versus passive.

The second theme, *personalization of treatment*, had 3 subthemes:

1. Flexibility and autonomy.
2. Stigma and privacy.
3. Functionality, content, and interface.

The third theme, *the value of receiving personal support in DHIs*, had 4 subthemes:

1. Support for understanding DHIs.
2. Support to enhance commitment and motivation.
3. Suitability and desire for additional support.
4. Support to develop a virtual therapeutic relationship.

### Theme 1: Initial Motivations and Approaches to Digital Health Interventions

Participants’ initial motivations and approaches to engaging with DHIs had a significant impact on the perceived helpfulness of the intervention. Those who approached the DHI with a sense of hope that it might be helpful and had an active, committed approach to see the treatment through had more positive experiences of treatment and reported greater benefits than those who were initially more cynical and utilized a passive approach in their engagement with DHIs. Thus, the participant approach to DHIs impacted the recruitment and therapeutic process. This began when participants contemplated participating in the study and continued throughout their engagement with the DHI. This is explored further in the following subthemes.

#### Initial Motivations: Hope, Accessibility, and Cynicism

A total of 15 papers reported on participant expectations of participating in a DHI trial [34-48]. Participants initially decided to engage with a DHI for several reasons. These included hope for recovery and the desire to improve health and reduce symptoms through self-management. The prospect of using DHIs encouraged participants to feel empowered and manage their health by taking responsibility. Participants highlighted that participating in a DHI would enable them to develop coping strategies to manage their difficulties and increase their self-efficacy. DHIs provided a sense of agency to move from a passive to an active role in managing their condition.

DHIs were also viewed as novel approaches to treatment, which provided an alternative opportunity to receive help. DHIs were perceived to be more easily available because they increased accessibility, flexibility, and choice in accessing therapy:

I think it’s an easy way to access help without having to like – you know it’s easy, it fits into your lifestyle, it’s convenient.

48

There were some negative expectations about DHIs, including skepticism about its helpfulness and concerns about whether a therapeutic relationship could be established remotely. However, in some cases, the ambivalence was overturned once participants commenced treatment, and there was surprise at how quickly a relationship could be established remotely.

#### Participant Approaches to Engaging With a Digital Health Intervention: Active Versus Passive

Participants’ approach to DHIs and technology in general affected motivation to continue engaging with DHIs. This was reported in 17 papers [24,34,37-39,41,44-46,48-55]. Participants who took a more active approach could see the unique benefits of DHIs over medication or face-to-face therapy:

Rather than just saying well here's your pills or sit there and talk to somebody for 35 minutes…actually felt like I was doing something to help myself.

54

Participants with an active approach embraced independent work. This involved actively processing information received (eg, taking time to reflect on the sessions), educating themselves about their condition, and applying the learning to their daily living.

Engaging with the DHI gave participants a sense of empowerment, understanding, and awareness about their condition and its triggers, which encouraged them to utilize the tools for self-management. It gave participants a sense of accomplishment and provided greater understanding, and participants felt “inspired to take more control” [38].

This theme was strongly represented in Bendelin et al [49], who identified that an active, self-reliant approach to treatment was related to outcomes that were more positive. However, participants who had a passive style of working struggled to apply the treatment and were more likely to discontinue treatment.

With regard to completing therapy, participants with an active approach felt a sense of obligation or personal commitment to complete the therapy because they had agreed to participate in the DHI and owed it to the researcher or research team to complete the treatment. Others reported that they completed sessions because they valued the importance of research.

Participants with a more passive approach, however, struggled to maintain motivation. They found the nature of DHIs to be “quite difficult,” “quite stressful” [52], and isolating. They preferred face-to-face sessions and felt that this was an essential component of personalized practical and emotional support.

### Theme 2: Personalization of Treatment

The degree and ways in which DHIs were personalized to participants’ situations and health status were deemed to impact the value of the treatment. The flexibility and convenience of DHIs had differential effects. For some participants, this made it more accessible and possible for them to engage in treatment in a way that traditional approaches could not. However, for others, the lack of structure, protected time, and accountability, present in more formal face-to-face therapy, meant that they forgot to complete sessions or disengaged from DHIs. Stigma and privacy were also a double-edged sword: for some, the anonymity of DHIs helped them to trust the process and engage; for others, the lack of a separate, private space to engage with difficult issues felt unsafe. There was broad agreement that DHIs with a simple interface and succinct content were preferred. There was also consensus that reminders, feedback on progress, and acknowledgment of achievements helped to support engagement.

#### Flexibility and Autonomy of the Digital Health Interventions

Flexibility and autonomy were emphasized in the majority of papers, with 19 papers reporting the flexible and autonomous nature of DHIs [24,35-42,45-49,52-56]. Some participants perceived DHIs to be more accessible and flexible. This led participants to feel that they had more autonomy and choice over their treatment. They used DHIs more responsively when they needed it, and this positively impacted treatment completion:

I like the fact that I could do it in my own time at home...cause I have three children so it wasn't like I would have to try to have to make appointments and get child care so I could do it when they were in bed or you know whenever it sort of seemed to fit in with my lifestyle I suppose.

56

Conversely, for some participants, DHIs lacked the structure and protected the treatment time they wanted, which subsequently impacted the motivation to complete treatment. Where interventions were self-guided and did not include monitoring, participants felt less obligated to complete sessions, particularly if they had competing priorities.

#### Stigma and Privacy

DHIs appealed to some participants because they were perceived to reduce the stigma and anxiety associated with seeking psychological help for mental health conditions. This was reported in 13 papers [35,36,41,42,44,48,49,51-53,55-57]. For participants who had not accepted their condition or felt afraid to express their thoughts, DHIs provided a safe platform to access support from the comfort of their own home. Participants felt more comfortable expressing their feelings on a computer rather than face-to-face because the DHI felt more private. Participants felt less judged and were able to be more honest and open in expressing their feelings to a computer rather than a person. They liked that they “didn’t have to tell anybody else face to face,” and found the DHI to be “a way of coping privately but in a structured way”’ [36]. However, some participants had concerns about the security and privacy of data [51]. Some believed that a trusting and therapeutic relationship could only be formed face-to-face.

#### Functionality, Content, and Interface

There was great variability in the DHIs used, consisting of different interventions and varying levels and forms of support. Themes related to DHI functionality, content and interface were highlighted in 21 papers [24,34-39,41-48,50-52,55-57]. Participants reported that content simplicity, reminders, and progress monitoring were very important aspects of functionality, the absence of which impacted treatment completion and satisfaction. This is because it influenced user identification with the material and provided feedback. Accessibility on a range of platforms, content relevance, and ease of navigation, readability, and inclusion of interactive elements impacted user acceptability and engagement with DHIs.

### Theme 3: The Value of Responsive Personal Support

This theme was identified in 24 of 25 papers. Only Kuhn et al [39] did not report this. Participants were able to seek treatment to help them self-manage their symptoms via the use of DHIs, but they still valued some form of human, responsive, personal support even if this support was not provided face-to-face. The key elements of additional support valued by participants were support that was personal and human and support that was rapidly responsive to their emotional state, personal difficulties, and achievements. Participants identified that additional support in DHIs helped them better understand DHIs, increased commitment and motivation, and helped form a more therapeutic engagement with DHIs. The rapidly responsive contact with a supporting person or therapist seemed to be missing from those who had poorer experiences of DHI. The presence and value of the provision of some form of personal support were identified to be integral in the majority of studies and forms the most influential theme.

#### Support for Understanding Digital Health Interventions and Treatment

Incorporating some form of support in DHIs aided participants’ understanding of the purpose of the intervention. This was particularly pertinent where participants were ambivalent about participating in a DHI or were unsure about the need or value of receiving psychological support. This was emphasized in 16 papers [24,35-38,40,44-48,51,52,54,55,57]. Where support was not provided, participants misunderstood the difference between a research trial and the DHI and would often assume that trial participation was part of therapy. Guided support provided participants with direction about the interpretation of the treatment session content and made therapy more personally relevant.

#### Support for Enhancing Commitment and Motivation

Incorporating some form of support to enhance commitment and motivation was highlighted in 17 papers [35,37,38,41,43-51,53-55,57]. Owing to the autonomous nature of DHIs, participants reported forgetting or feeling less obligated to engage in treatment compared with face-to-face therapy, as highlighted in the previous theme. Without additional support, they struggled to relate to and apply the therapy content to their own condition, leading to disengagement from the DHI. Receiving feedback from a therapist or others allowed participants to monitor their progress, prevented forgetfulness, and encouraged participants to continue with therapy. Some form of communication was helpful and was achieved via a number of mediums, including face-to-face and remotely via emails, phone calls, and SMS text messages. Thus, receiving support facilitated understanding of symptoms, prevented forgetfulness to complete modules, and provided encouragement to overcome challenges and reduced isolation. Disengagement from a DHI was more likely in the absence of support as it reduced commitment and motivation to complete therapy.

From my point of view, the contact with the therapist was an essential aspect of therapy. Therefore, I lost all my interest in the therapy and didn't want to continue.

43

#### Suitability and Desire for Additional Support

Questions regarding the suitability of DHIs for some problems were raised in 12 papers, alongside some patients’ desire for additional responsive support when DHIs became challenging or unsuitable [24,34,43,45,46,50-56]. DHI therapy sessions could be physically and mentally exhausting, and for some participants, it exacerbated symptoms of low mood and anxiety. Therefore, some participants wanted additional support to manage these negative feelings. The absence of support made module completion overwhelming for some participants, leading them to prioritize other commitments or discontinue the treatment.

Hind et al [52], focusing on DHIs for people with depression and physical comorbidities, found that completion of DHI sessions placed physical demands on participants. Where support was not provided, some form of support was recommended to overcome feelings of isolation and enable emotional expression [34,51,52]. This subtheme was not highlighted by any of the papers that included face-to-face support [24,33,35-38,44,57].

#### Support for Developing a Virtual Therapeutic Relationship

The interpersonal and relational aspects remained an essential ingredient of therapy, even if it was delivered as a DHI. This subtheme was reported in 18 papers [24,33-35,37,38,41,43-48,50,51,53-55]. Participants who engaged in DHIs reported feeling surprised at how quickly a relationship could be formed remotely with a person. They valued expressing feelings in written form because it enabled self-reflection and communication of emotion without interruptions. Attributes associated with developing a therapeutic relationship face-to-face were also identified in the DHIs. This included building a positive relationship between trust and feeling understood. Participants referred to the therapist as “a real friend” and likened the DHI to a “face-to-face session” [24].

Participants who disengaged from DHIs found them to be impersonal and expressed a preference for face-to-face therapy. The absence of visual cues such as eye contact and gestures was perceived to reduce emotional closeness, and made participants question whether the therapist was giving them their undivided attention. The use of written communication methods and the associated time delay between responses were seen as barriers to developing a therapeutic relationship. The absence of face-to-face contact was perceived to lack empathy, be machine-like *“*cold, not very friendly” [35], and this negatively impacted the therapeutic relationship.

### Summary of Meta-Synthesis

The themes emerging from the meta-synthesis acknowledge the variability within individuals’ experiences of DHIs for depression, anxiety, and somatoform disorders. DHIs were perceived both positively and negatively by participants, and this was influenced by the participants’ expectations and needs. Personalization was an overarching theme; participants who had a preference for flexibility and autonomy perceived these to be the benefits of DHIs. In addition, the functionality of the DHIs was also important to participants. The key means of personalizing treatment identified as helpful by participants was the addition of rapid, responsive personal or human support. Thus, DHIs need to be responsive to participant preferences and experiences.

## Discussion

### Principal Findings

This systematic review aimed to meta-synthesize qualitative studies exploring the views of people who had been invited to participate in DHIs for depression, anxiety, and somatoform disorders. The first aim of the review was to examine service user perceptions of DHIs concerning their acceptability and usability. Findings from the meta-synthesis indicate that the acceptability and usability of DHIs differ on the basis of initial personal perceptions of DHIs, associated motivations, and the degree of responsive support offered. Participants’ perceptions of DHIs can be positive or negative depending on expectations, preferences, and approaches to DHIs. Some participants felt more comfortable expressing their feelings on a computer rather than sharing it with somebody face-to-face because the DHI felt more private. Other participants found the nature of DHIs to be impersonal and preferred face-to-face therapy. This did not differ by DHI type or format, suggesting that the acceptability and usability of DHIs is influenced by individual perceptions and preferences. This study highlights that therapeutic work in DHIs is a dynamic process and is perceived positively or negatively depending on how well the DHI is adapted to the participants’ preferences. The personalization of DHIs was an overarching theme, implying that DHIs need to consider individual preferences, circumstances, and needs to improve therapy completion rates for DHIs.

The second objective of the review was to examine aspects of DHIs that are valued and work well and those that could be improved or altered. The third aim of the review was to explore why service users chose to use, continue with, or stop using DHIs. This meta-synthesis emphasizes the significance of receiving personal support in DHIs and is consistent with the findings of O’Connor et al [26] and Knowles et al [27], both highlighted the need for personalization and availability of support in DHIs. The meta-synthesis also informs some of the research priorities identified by Hollis et al [25]. This study highlights that the suitability of DHIs is based on differing needs and that DHIs could be optimized by incorporating additional support. This systematic review extends on previous findings of Knowles et al [27] by demonstrating that personalized support was valued across studies irrespective of DHI type, format, or disorder. Participants who received self-guided DHIs expressed dissatisfaction with the lack of human interaction and expressed that it was required to increase commitment as it enabled personal support and feedback. Participants who received face-to-face contact as part of the DHI expressed that incorporating interpersonal features such as the provision of support was central because it personalized therapy. Personalized support facilitated an understanding of therapy, increased commitment and motivation to continue treatment, and helped form a more therapeutic engagement with DHIs. Similarly, participants in DHIs consisting of email or phone support reiterated that the presence of a supporter personalized therapy. However, they also expressed a desire for more contact with a supporter. Participants in the 2 studies that provided no therapy and only technical support or reminders [50,54] expressed a need for personalization in the form of feedback and emotional support. The functionality of DHIs was perceived to facilitate or hinder engagement. DHIs that were easily accessible and interactive were viewed as more beneficial than DHIs that were harder to navigate and inaccessible on a range of platforms. DHIs consisting of smartphone apps were perceived to be easily accessible. A simple interface and succinct content with reminders, feedback on progress, and acknowledgment of achievements also helped to support the completion of therapy.

This review also highlights that the flexible and autonomous nature of DHIs and the desire to gain greater awareness to self-manage conditions can affect participants’ choice of whether to engage with a DHI or not. These aspects are consistent with a previous systematic review on DHIs [26], which also emphasized the significance of personal agency and motivation to improve awareness and self-management of health when considering recruitment to DHIs. The current meta-synthesis enhanced these findings by highlighting how individual expectations and perceptions influenced participant engagement and approaches to DHIs. Participants with an active approach perceived DHIs to be more favorable compared with face-to-face therapy and were more likely to actively engage with DHIs by reflecting on and applying session content to their daily lives. Subsequently, these participants were more likely to complete treatment compared with participants who found DHIs isolating. DHIs attracted participation when they were perceived to be accessible and tailored to individual needs. Participants could access treatment from their own homes, which was more convenient and appropriate for those who would otherwise not access treatment. DHIs were also perceived to be more appealing because they reduced the stigma of accessing treatment. However, for some participants, the autonomous nature of DHIs made it easier to prioritize other tasks and disengage from treatment because the sense of commitment to treatment was reduced.

The meta-synthesis illustrates the different requirements of support that can potentially be provided in a number of ways. Thus, on the basis of participant preferences and needs, DHIs could be tailored to meet individual presentations. DHI functions and level of support likely to be required could be determined by initially assessing participant expectations and needs, as opposed to uniformly offering DHIs as an all-or-nothing option.

### Strengths and Limitations

This is the first meta-synthesis to our knowledge that has looked at the acceptability and usability of DHIs across different types and formats and across a range of depression and anxiety disorders. This systematic review included all DHIs regardless of the delivery method (ie, text-based, automated, blended therapy), so it compared very different experiences. The themes emerging from the meta-synthesis highlight that personalized support was valued across studies irrespective of DHI type, format, or health condition. In particular, some form of human interaction was valued because it personalized therapy and increased motivation to complete therapy. This supports the generalizability of this finding, given that participants reported similar themes from a range of DHIs.

A limitation of including a range of different interventions is that some comparisons may have been incompatible or inappropriate across these rather different technologies. However, given the broad inclusion criteria, it is particularly important that personal support was still highlighted as a theme across studies and gives greater weight to its importance. We excluded peer-to-peer networks and DHIs of social support via the internet, use of social media, online support groups, or DHIs consisting of group therapy. Future research could explore whether participants’ experiences of these delivery formats differ or are similar to our findings. It is worth highlighting that technological competence was only identified in 3 studies [24,41,51] as a potential barrier to engaging with a DHI. This could be because the views captured in the papers are mainly of participants who chose to engage in a DHI and do not capture the experiences of participants who decided not to participate in a DHI. The majority of the DHIs were CBT-based despite including a broad range of mental health conditions, treatment settings, and types of DHIs. Recommended therapies, such as interpersonal therapy for depression, were notably absent from studies included [58]. In terms of DHI variability, the use of videoconferencing to provide therapy was only included in 1 study [24] and could be investigated further in the future. Videoconferencing has the potential to provide real-time face-to-face therapy remotely, thus improving accessibility but also increasing the level of personalized support. A recent RCT investigating the clinical and cost-effectiveness of videoconferencing CBT delivered to repeat unscheduled care users with health anxiety found completion rates to be substantially higher than other DHI studies [7]. Recent years have also seen an increase in the use of smartphone apps for the treatment of depression and anxiety and the use of VR and interactive games for the treatment of anxiety and phobias. Therefore, it will be useful to update this systematic review to incorporate research regarding the acceptability and usability of smartphone apps to ascertain whether similar themes emerge.

This systematic review was not carried out using double screening, with only 30% of data being extracted by both reviewers. However, the high level of congruence found for the subset sample implied that the screening methods were rigorous. The systematic review only included papers published in English, which may reflect the fact that the majority of the studies were conducted in European and American countries. However, major sources of technology production are found in non–English speaking countries, for example, China, Japan, and India. The majority of studies were for the treatment of depression, and there were no studies on the use of DHIs for the treatment of somatoform disorders; therefore, the conclusions might not apply to somatoform disorders. We excluded participants with bipolar disorder despite depression being a characteristic feature of bipolar disorder; hence, we were unable to see whether the findings could translate to this condition.

### Implications and Recommendations

On the basis of our findings from the meta-synthesis, we propose the following implications and recommendations to policy makers and commissioners:

1. Expectations and pre-existing beliefs about DHIs and their effectiveness can have an impact on patient experience and engagement. Therefore, addressing these expectations before beginning a DHI or building this into the initial stages of DHIs would help manage any misconceptions and address early barriers. This might also explain the high dropout rate from DHIs if people perceive them to be less effective than traditional approaches. An initial assessment should also include addressing patient preferences in terms of autonomy, level of support, and medium of communication. This would help identify whether additional support is needed, and if so, the level required. Responsiveness to these kinds of unnecessary barriers could aid engagement and retention in DHIs.
2. Personalization of DHIs and personalized support was an overarching element that ran throughout all themes. Communication of some form was valued by the majority of the participants. It clarifies the intervention’s purpose, personalizes therapy, and increases self-discipline and motivation to engage with DHIs. Thus, engagement with DHIs can be enhanced by including personal reminders for therapeutic activities and giving participants’ individualized feedback on their progress with therapeutic tasks. Ensuring that the interface and content is succinct and easy to navigate around would also likely reduce dropout from DHIs. Thus, future DHIs need to consider how feedback and reminders can be incorporated and presented.
3. Both the previous recommendations and many other individual problems described by participants in this study would be addressed by making additional support available to those using DHIs. There is a clear and consistent theme of preferred qualities for this additional support, particularly where the person is passive or indifferent in their willingness to engage with a DHI. Additional support should be personalized, incorporate some form of human interaction, and be rapidly responsive. This kind of support was especially desirable when participants met significant barriers to DHIs. Therefore, treatment engagement, experience, and response should be monitored throughout DHIs, so those who do not respond or have a poor experience can receive an appropriate level of additional support quickly.

### Conclusions

This review indicates that addressing patients’ initial expectations of DHIs could help improve uptake, therapy completion, and effectiveness. Furthermore, the addition of rapid, responsive personal and human support, albeit offered remotely, could improve patient experience of DHIs, particularly when patients find DHIs challenging. The recommendations offered by this review have the potential to improve recruitment and retention rates to participation in DHIs and guide the design of DHIs so that they are personalized and improve overall patient experience.

## Appendix

Multimedia Appendix 1 PRISMA 2009 checklist.

Multimedia Appendix 2 ENTREQ checklist.

Multimedia Appendix 3 Search strategy for MEDLINE.

Multimedia Appendix 4 CASP Quality appraisal of included studies.

Multimedia Appendix 5 Summary of included studies.

Multimedia Appendix 6 Sample characteristics of included studies.

Multimedia Appendix 7 First, second, and third order constructs and sub-themes and contrasting positive and negative participant experiences.

## Abbreviations

CASP: Critical Appraisal Skills Program
CBT: cognitive behavioral therapy
cCBT: computerized cognitive behavioral therapy
DHI: digital health intervention
GAD: generalized anxiety disorder
NIHR: National Institute for Health Research
OCD: obsessive-compulsive disorder
PRISMA: Preferred Reporting Items for Systematic Reviews and Meta-Analyses
PTSD: posttraumatic stress disorder
RCT: randomized controlled trial
VR: virtual reality
