# Supplementary material for: The Acceptability and Usability of Digital Health Interventions for Adults With Depression, Anxiety, and Somatoform Disorders: Qualitative Systematic Review and Meta-Synthesis
Source: J Med Internet Res. 2020 Jul 6;22(7):e16228. doi: 10.2196/16228 (PMC7381032; doi:10.2196/16228)
Supplement: Multimedia Appendix 3 [file jmir_v22i7e16228_app3.docx]

**Multimedia Appendix 3 Medline Search strategy**

Database: Ovid MEDLINE(R) and In-Process & Other Non-Indexed Citations <1946 to July 17, 2018>

Search Strategy:

--------------------------------------------------------------------------------

1 exp TELEMEDICINE/ (22975)

2 exp INVENTIONS/ (1284)

3 (eHealth or ehealth* or e-health* or e health* or "electronic adj health" or e-mental health or m-mental health or mhealth* or m-health* or "mobile health*" or "m health" or ePsych* or e-Psych* or (electronic adj psyc*) or eTherap* or e-therap* or (electronic adj therap*)).tw. (6798)

4 ("telebehavio?ral health" or "tele care" or telecare or "tele coaching" or telecoaching or telecomm* or tele-comm* or "tele conference*" or teleconference* or "tele consultation" or teleconsultation or "tele health care" or "tele health*" or telehealth* or tele-health or "tele management" or telemanagement or "tele med*" or tele-med* or "tele mental health*" or "telemental health*" or telemetry or tele-monitor* or telemonitor* or telepractice or "tele practice" or tele-psych* or telepsych* or "tele speech" or telespeech or "tele therap*" or tele-therap* or teletherap*).tw. (17342)

5 ((intervention* or invention* or innovation*) and technolog*).tw. (31791)

6 exp Mobile Applications/ (3126)

7 ((app or apps or application) adj2 (smartphone* or smart-phone or mobile* or phone* or sensor* or software)).tw. (5513)

8 exp Video Games/ (4246)

9 (gaming or gamification or videogam* or computer gam* or video gam* or electronic gam*).tw. (6025)

10 exp Videoconferencing/ (1536)

11 (videoconferenc* or "video conferenc*" or videoconsultation* or "video consultation*" or "video technolog*" or "video model*" or Skype* or facetime or webex).tw. (3018)

12 wearable electronic devices/ or fitness trackers/ (513)

13 (smartwatch* or (wearable adj device*) or wearables or "real-time monitoring device*" or actigraphy or accelerometer*).tw. (13874)

14 exp Virtual Reality/ or exp Virtual Reality Exposure Therapy/ (744)

15 ("virtual reality" or "augmented reality").tw. (7825)

16 ("interactive multimedia" or "interactive software").tw. (558)

17 ("digital media" or "software program*").tw. (4714)

18 ((Internet or digital* or online* or on-line or web* or virtual or computer*) adj2 (deliver* or information or communication* or assisted or e-learning or support)).tw. (37043)

19 (ipad adj2 (app or apps or application or intervention*)).tw. (125)

20 (Internet adj2 based).tw. (7287)

21 (technolog* adj2 (deliver* or information or communication*)).tw. (17182)

22 ("interactive technolog*" or "wearable technolog*" or "mHealth technolog*" or "mobile technolog*" or "sensor technolog*").tw. (2925)

23 *Robotics/ (13880)

24 (robot or robots or robotics).tw. (17761)

25 or/1-24 (185686)

26 exp DEPRESSION/ (102399)

27 exp Depressive Disorder/ (99396)

28 exp Anxiety Disorders/ (74274)

29 exp ANXIETY/ (74098)

30 exp Somatoform Disorders/ (18154)

31 exp HYPOCHONDRIASIS/ (2252)

32 exp Dysthymic Disorder/ (1106)

33 exp Conversion Disorder/ (2705)

34 exp Body Dysmorphic Disorders/ (799)

35 exp Factitious Disorders/ (2962)

36 exp Stress Disorders, Post-Traumatic/ (28307)

37 exp Agoraphobia/ or exp Panic Disorder/ (7857)

38 exp Phobia, Social/ (375)

39 exp Obsessive-Compulsive Disorder/ (13391)

40 exp Stress Disorders, Traumatic, Acute/ (427)

41 exp Premenstrual Dysphoric Disorder/ (85)

42 exp Mutism/ (1020)

43 exp Mood Disorders/ (111205)

44 exp Cyclothymic Disorder/ (644)

45 exp NEUROTIC DISORDERS/ (17934)

46 exp Phobic Disorders/ (10663)

47 exp Adjustment Disorders/ (4111)

48 (depress* or anxiety or anxieties or anxious or GAD or somatiz* or somatis* or somatic or somatoform* or multisomat* or multi somat* or dysthymic disorder* or conversion disorder* or pain disorder* or hypochondria* or body dysmorphi* or factitious disorder* or panic disorder* or panic attack* or phobi* or agrophobi* or obsessive compulsive* or OCD or post traumatic stress* or PTSD or stress disorder* or disruptive mood dysregulation disorder* or body dysmorphic disorder* or premenstrual dysphoric disorder* or mutism or mood affective disorder* or mood disorder* or cyclothymi* or neurotic disorder* or adjustment disorder* or medically unexplained symptom*).tw. (625983)

49 or/26-48 (721287)

50 25 and 49 (7625)

51 exp QUALITATIVE RESEARCH/ (39832)

52 exp INTERVIEW/ (27980)

53 exp Focus Groups/ (24746)

54 (("semi-structured" or semistructured or unstructured or informal or "in-depth" or indepth or "face-to-face" or structured or guide or guides or follow up or self report) adj3 (interview* or discussion* or questionnaire*)).tw. (115410)

55 (focus group* or advisory group* or qualitative or ethnograph* or fieldwork or field work or key informant or thematic analy* or grounded theor* or phenomenolog* or discourse analy* or content analy* or narrative* or observational method* or open ended evaluation* or action research or inductive analy* or emic or etic or hermeneutic* or constant compar* or grounded theor* or lived experience* or life experience* or theoretical sampl* or purposive sampl* or quasi-experiment* or (case adj2 stud*)).tw. (465420)

56 exp "Surveys and Questionnaires"/ (903821)

57 exp Self Report/ (23304)

58 (field note* or fieldnote* or field record* or field stud* or structured categor* or unstructured categor* or (participant* adj3 observ*) or (nonparticipant* adj3 observ*) or (non participant* adj3 observ*)).tw. (23991)

59 or/51-58 (1414813)

60 50 and 59 (2476)

61 limit 60 to english language (2430)
