# Supplementary material for: The Acceptability and Usability of Digital Health Interventions for Adults With Depression, Anxiety, and Somatoform Disorders: Qualitative Systematic Review and Meta-Synthesis
Source: J Med Internet Res. 2020 Jul 6;22(7):e16228. doi: 10.2196/16228 (PMC7381032; doi:10.2196/16228)
Supplement: Multimedia Appendix 4 [file jmir_v22i7e16228_app4.docx]

| Reference | Q1^a^ | Q2^b^ | Q3^c^ | Q4^d^ | Q5^e^ | Q6^f^ | Q7^g^ | Q8^h^ | Q9^i^ | Q10^j^ |
| --- | --- | --- | --- | --- | --- | --- | --- | --- | --- | --- |
| Advocat 2010 | Y^k^ | Y | Y | U^l^ | Y | Y | Y | U | U | Y |
| Ashford 2018 | Y | Y | Y | Y | Y | Y | Y | U | U | U |
| Bauer 2018 | Y | Y | Y | Y | Y | N^m^ | Y | Y | Y | Y |
| Beattie 2009 | Y | Y | Y | Y | Y | N | Y | Y | Y | Y |
| Bendelin 2011 | Y | Y | Y | U | Y | Y | Y | Y | Y | Y |
| Clarke 2016 | Y | Y | Y | U | Y | N | Y | Y | Y | Y |
| Donkin 2012 | Y | Y | Y | Y | Y | Y | Y | Y | Y | Y |
| Etzelmueller 2018 | Y | Y | U | U | Y | N | Y | U | Y | Y |
| Farzanfar 2007 | Y | Y | U | U | U | N | Y | U | N | U |
| Fernandez -Alvarez 2017 | Y | Y | U | U | Y | Y | Y | Y | Y | Y |
| Gega 2013 | Y | Y | Y | Y | Y | Y | Y | Y | Y | Y |
| Gerhards 2011 | Y | Y | Y | Y | Y | N | Y | Y | Y | Y |
| Hind 2010 | Y | Y | Y | Y | Y | N | Y | Y | Y | Y |
| Holst 2017 | Y | Y | Y | Y | Y | Y | Y | Y | Y | Y |
| Johansson 2015 | Y | Y | Y | Y | Y | Y | Y | Y | Y | Y |
| Knopp Hoffer 2016 | Y | Y | Y | U | Y | Y | Y | Y | Y | Y |
| Knowles 2015 | Y | Y | Y | Y | Y | Y | Y | Y | Y | Y |
| Kuhn 2014 | Y | Y | N | N | N | N | Y | N | N | Y |
| Lillevoll 2013 | Y | Y | U | U | Y | U | Y | Y | Y | Y |
| Lovell 2017 | Y | Y | Y | Y | Y | Y | Y | Y | Y | Y |
| Ly 2015 | Y | Y | Y | U | Y | Y | Y | Y | Y | Y |
| Pugh 2015 | Y | Y | N | U | U | U | U | Y | Y | Y |
| Purvez 2013 | Y | Y | Y | Y | Y | Y | Y | Y | Y | Y |
| Richards 2016 | Y | Y | U | U | U | U | U | U | Y | Y |
| Walsh 2017 | Y | Y | Y | U | Y | U | U | Y | Y | Y |
| Wilhelmson 2013 | Y | Y | Y | Y | Y | Y | Y | Y | Y | Y |

^a^Q1 Was there a clear statement of the aims of the research?

^b^Q2 Is a qualitative methodology appropriate?

^c^Q3 Was the research design appropriate to address the aims of the research?

^d^Q4 Was the recruitment strategy appropriate to the aims of the research?

^e^Q5 Was the data collected in a way that addressed the research issue.

^f^Q6 Has the relationship between researcher and participants been adequately considered?

^g^Q7 Have ethical issues been taken into consideration?

^h^Q8 Was the data analysis sufficiently rigorous?

^i^Q9 Is there a clear statement of findings?

^j^Q10 How valuable is the research?

^k^Y=yes ^l^U=can’t tell ^m^N=no

Reference: CASP. Critical Appraisal Skills Programme (CASP). 2015. URL: http://www.casp-uk.net/ [accessed 2018-11-15]
