# Supplementary material for: The Acceptability and Usability of Digital Health Interventions for Adults With Depression, Anxiety, and Somatoform Disorders: Qualitative Systematic Review and Meta-Synthesis
Source: J Med Internet Res. 2020 Jul 6;22(7):e16228. doi: 10.2196/16228 (PMC7381032; doi:10.2196/16228)
Supplement: Multimedia Appendix 5 [file jmir_v22i7e16228_app5.docx]

| No of studies (%) | |
| --- | --- |
| Country  England  Republic of Ireland  Sweden  Germany  Spain  Norway  Netherlands  Australia  America  Canada | 7 (29)  1 (4)  4 (17)  1 (4)  1 (4)  1 (4)  2 (8)  3 (13)  3 (13)  1 (4) |
| Setting  Community  Primary Care  Psychiatric Services  Medical Clinics | 10 (42)  7 (29)  6 (25)  1 (4) |
| Condition  Depressive disorders  Postpartum depression  Comorbid cardiovascular and depression Comorbid multiple sclerosis and depression  Anxiety  Panic disorder  Post-partum anxiety  Generalised anxiety disorder  Obsessive Compulsive Disorder  Post-Traumatic Stress Disorder  Depressive and anxiety disorder  Depression or (mixed depression and anxiety)  Depression, anxiety and/or stress symptoms | 11 (46)  1 (4)  1 (4)  1 (4)  1 (4)  1 (4)  1 (4)  1 (4)  1 (4)  1 (4)  2 (4)  1 (1)  1 (1) |
| Data Collection  Individual interviews  Focus group and individual interviews  Focus groups  Individual interviews and written based free text responses  Free text responses | 18 (75)  1 (4)  1 (4)  2 (8)  2 (8) |
| Additional support provided  None  Email/phone/text  Face-to-face  DHI Platform  Desktop based computer  Smart phone application  Mobile phone and web based  Blended approach  Computer telephony system  Treatment approach  CBT principles  Transdiagnostic  Behavioural activation  Collaborated care model  Medication adherence and self-care training | 5 (21)  13 (54)  6 (25)  19 (79)  2 (8)  1 (4)  1 (4)  1 (4)  19 (79)  1 (4)  1 (4)  1 (4)  2 (8) |
