# Supplementary material for: The Acceptability and Usability of Digital Health Interventions for Adults With Depression, Anxiety, and Somatoform Disorders: Qualitative Systematic Review and Meta-Synthesis
Source: J Med Internet Res. 2020 Jul 6;22(7):e16228. doi: 10.2196/16228 (PMC7381032; doi:10.2196/16228)
Supplement: Multimedia Appendix 6 [file jmir_v22i7e16228_app6.docx]

| **Ref** | **Aims** | **Sample (age, gender, ethnicity)** | **Study setting** | **Condition** | **Type of DHI** | **Support provided** | **Data collection point** | **Data collection** | **Data analysis** | **RCT** |
| --- | --- | --- | --- | --- | --- | --- | --- | --- | --- | --- |
| **Advocat 2010** | Explore experiences of internet delivered CBT | 2 M, 8 F,  Age range  20-66 years  9 Anglo-Australia,  1 Chinese-Australian | Community  Australia | Panic Disorder | Internet delivered CBT for Panic Disorder | Yes, email support | Trial completion | Face to face in depth interviews | Open coding using a two-step model | Yes |
| **Ashford 2018** | Investigate the feasibility and acceptability of a web-based treatment with telephone support | 5 F interviewed and 31 comments Mean age  32.02 age range 22-43 years  84/89 (94%) Caucasian (whole sample) | Community England | Post-Partum women with anxiety | 9 module Web based self- guided treatment based on Cognitive Behavioural and mindfulness principles | Yes, optional weekly phone support | Trial completion or withdrawal | Phone interview or web-based survey plus optional comments written after each module | Inductive thematic analysis | Yes |
| **Bauer 2018** | Mixed methods study to assess feasibility and acceptability of a mobile health platform supporting collaborative care | 7 M, 10 F  16 White  Age range  18-65+  16 (94%) White/  Caucasian | Primary Care  America | Depression or anxiety | Collaborative care model using a smart phone app and web-based provider dashboard | Yes, care manager input | Four weeks after app installation | Open ended semi-structured phone interview | Directed content analysis to develop priori codes | No |
| **Beattie 2009** | Explore expectations and experiences of online CBT | 17 F, 7 M  Age range  24-66 Ethnicity not stated | Primary Care  England | Depression | Live CBT delivered via the internet | Yes, psychologist via instant messaging | Before and after treatment | Repeated semi-structured interviews | Thematic approach drawing on constant comparative method | No in parallel |
| **Bendelin 2011** | Explore views of internet guided self-help treatment | 6 F, 6M  Age range  20-62 years, mean age 36  3 All Native Swedes | Community  Sweden | Depression | Internet based CBT Guided self-help and email therapy | Yes, via email | Within 8-10 months of treatment completion | In depth face to face interviews | Thematic analysis and grounded theory | Yes |
| **Clarke 2016** | Explore therapeutic alliance with a fully automated mobile phone and web based intervention | 13 F, 3 M  Mean age  40.1 years  Ethnicity not stated | Community  Australia | Depression/ anxiety/  Stress | Self-guided fully automated CBT based mobile phone and web based intervention | No | Trial completion | Semi-structured interviews | Thematic analysis | Yes |
| **Donkin 2012** | Explore the motivators that influence persistence to continue online therapies | 14 adults aged over 45 with physical health morbidities  No other information provided | Community  Australia | Co-morbid cardiovascular and depressive symptoms | 12 week fully automated online CBT intervention for depression | Some, reminder email sent If module not complete telephone call made by RA with scripted reminder , No therapy provided | 6-12 month follow up stage | 10 phone and two f2f semi-structured interviews | Grounded theory approach using theoretical coding | Yes |
| **Etzelmueller 2018** | Evaluate patient experience of blended CBT | 9 F, 6 M  Median age  58.94, age range 53-64 years old  Ethnicity not stated | Mental Healthcare  Germany | Major depressive disorder | Blended internet and video based therapy sessions plus online and smart phone based monitoring | Yes therapist | Treatment completion | Semi-structured telephone interviews | Framework method Thematic analysis | No |
| **^b^ Farzanfar 2007** | Explore attitudes of patients using an automated telephony system | 9 F, 6M  2 Hispanics,  5 Blacks and 8 Whites Age range 20-60 | Psychiatric Clinics  America | Depression | Telephone Linked Communications (TLC) for Depression. An automated telephony system that asks questions and provides information and counselling | Yes, psychiatric Clinic Appointments | After each week of using the system | Three weekly depth interviews | Thematic analysis | Before RCT |
| **Fernandez-Alvarez 2017** | Explore experiences of patients dropping out of a trans-diagnostic online intervention | 8F, 2 M  Mean age 35.4 years, range 23-61 years Ethnicity not stated | Public mental health care system  Spain | Emotional disorders | Transdiagnostic internet-based treatment (IBT) consisting of 12 modules | Yes, minimal support from a therapist (5-10-minute weekly phone call) plus two weekly automated reminder text messages | Dropping out after completion of at least 3 modules | Semi- structured interview, 5 interviews via video-conference and 5 face to face | Consensual Qualitative Research (CQR) applied to analyse the narrative content | Yes |
| **Gega 2013** | Explore patient experiences of computerised CBT compared to therapist delivered CBT | 2 F, 3M  Age range  19-33 years  Ethnicity not stated | Primary Care  England | Depression or mixed depression and anxiety | Beating the Blues computerised CBT and therapist assisted CBT (f2f) | Yes, when receiving therapist assisted CBT  Ccbt – minimal therapist input f2f to provide technical help and progress review after each session | Trial completion or drop out from both modalities | Repeated semi-structured interviews | Thematic analysis using an inductive approach | No repeat case series |
| **Gerhards 2011** | Explore patient experiences with online self-help cCBT and explanations for low treatment adherence and effectiveness | 9M, 9 F  Mean age 43.6  ethnicity not stated | Primary Care  Netherlands | Depression | 8 weekly sessions of an online multimedia interactive program “Colour your Life” (CYL) for depression | No | Treatment completion or withdrawal | Semi-structured interviews | Inductive content analysis in line with grounded theory approach | Yes |
| **Hind 2010** | Investigated the acceptability of cCBT | 4 M, 13 F  Median age 46 (30-61) years  Ethnicity not stated | England  MS Clinic | Depression in people with MS | CCBT, 8 weekly sessions of Beating the Blues or 5 weekly sessions of Mood Gym | No | Written feedback after sessions. Interviews after first session, and withdrawal/ completion | Semi-structured interviews and written feedback | Framework analysis | Yes |
| **Holst 2017** | Explore Primary Care patient’s experiences of ICBT therapy | 7 F, 6 M Mean age 41 years range (27-68) years 12 Native Swedes | Primary Care  Sweden | Depression | Internet delivered self-help program consisting of interactive elements and a CD | Yes, minimal therapist contact in the form of a weekly email and 3 phone calls in total Additional contact provided if required | Trial completion | Semi-structured focus group and individual interviews | Systematic text condensation | Yes |
| **Johansson 2015** | Explore patients’ experience of non-adherence to ICBT | 6 F, 1M Mean age 39 3 years, range (21-69) Ethnicity not stated | Psychiatric setting  Sweden | GAD | 8 modules of Self-help internet delivered CBT | Yes, email based weekly support from a Clinical Psychologist Phone reminders made for non-completion of weekly modules | Completion of at least one and no more than 7 treatment modules | Semi-structured interviews | Grounded theory using constant comparative process | Yes |
| **Knowles 2015** | Explore patient experience of cCBT | 26 F, 10 M 34 (94%) White British  Mean age 51 (29-69) years 34 (94%) White British, 2 other White | Primary Care  England | Depression | CCBT, 6-8 weeks of Mood Gym or Beating the Blues | Yes, minimal, technical and general support via phone calls on a weekly basis Psychological therapy not provided | After completion of 4 months trial follow up | Semi-structured interviews | Inductive and deductive approaches using the constant comparative method Fragmenting and connecting | Yes |
| **Kuhn 2014** | Explore user perspectives if a smart phone app | 34 M (75.6%), 11 women Mean age  45. 25 years  46 7% Caucasian | Residential PTSD programmes  America | PTSD IN Veterans | PTSD Coach, a smart phone app based on CBT Principles | No | After using the app for several days | Focus groups | Grounded theory approach Data related to use of the app extracted from notes taken | No |
| **^a^Lillevoll 2013**  **^a^Wilhelmson 2013** | Explore pts experiences of being in ICBT | 5M, 9 F (64%)  Age range  22-61 years  Ethnicity not stated | Primary Care  Norway | Depression | Guided internet based treatment - Five sessions of Mood Gym | Yes, weekly f2f consultations with a therapist over a minimum of 7 weeks | Treatment completion and withdrawal | Semi-structured interviews | A phenomenological-hermeneutical analysis | Yes |
| **^a^Lovell 2017**  **^a^Knopp Hoffer 2016** | Explore user perspective of two low intensity CBT interventions | 21 F (58%), 15 M (42%)  Mean age 42.7 range  20.7-64.5 years  Ethnicity not stated | IAPT Mental Health Services  England | OCD | Two low intensity CBT interventions;  Guided self-help (self-help book + 6h professional support) and minimally supported CCBT (access to computer program plus 1 hour professional support) | Yes, guided self-help consisted of support from a psychological wellbeing practitioner (PWP) consisting of 1 hour initial session (f2or phone) and 10, 30 minute sessions over 12 weeks  Minimal supported consisted of 6, 10 minute brief telephone calls from a PWP | Between 4-18 months after entering trial 34 after treatment completion, two in treatment | Open-ended inductive interviews 54% over the phone and 46% f2f | Thematic analysis using constant comparison method | Yes |
| **Ly 2015** | Explore participant’s views of smartphone based behavioural activation treatment for depression | 6 F, 6 M Age range 21-59 years  Ethnicity not stated | Community Sweden | Depression | Guided behavioural activation self-help web –based treatment administered via a smart phone | Yes, minimal therapist contact (max time 20 minutes per week) via email or SMS like messages sent via the platform | 6 months after trial involvement | In depth phone interviews 5 had a positive experience, 3 negative and 4 neutral | Thematic analysis | Yes |
| **Pugh 2015** | Gain an understanding of patients’ experiences of therapist assisted internet based cognitive therapy | 24 (100%) F,  22 (92%) Caucasian  Age not stated | Community Canada | Postpartum depression | Therapy assisted internet based cognitive therapy (TAIBCT) | Yes, one e-mail a week from assigned internet therapist | Program completion | 10 open ended survey questions on a secure internet survey site | Thematic analysis | Yes |
| **^b^Purvez 2013** | Explore the experience of self-help cCBT to alleviate psychological distress | 6F and 1M Age range  30-57 years | Community  England | Depression | Self-help Ccbt program called Blues Begone Program sent on a DVD to be installed on a computer Interacts with pts through animated talking heads | No | After program completion | Semi-structured interviews | Interpretative Phenomenological  Analysis (IPA) | No |
| **Richards 2016** | Understand user experience of supported internet-delivered low intensity treatment for depressive symptoms | 281, 211 (75%) F, 70 (25%) M Mean age 38. 10  range 18-63 Ethnicity not stated | Community Ireland | Depression | Space from Depression – an internet delivered CBT intervention consisting of 8 modules | Yes, weekly reviews by a trained supporter providing guidance, support and motivation via email | Post treatment or completion of at least one module | Self-reported online questionnaire consisting of qualitative questions | Thematic analysis | Yes |
| **Walsh 2017** | Explore participants’ experiences with an internet-delivered treatment | 5 F, 2M  Mean age  22. 86, age range 18-34 years 5 Irish and remaining American/  Russian | University  Ireland | Anxiety | Computer based CBT based space from anxiety 6 week programme | Yes, minimal support from an online therapist Post session  5-10 minutes feedback | 6-8 weeks after trial completion | Semi-structured interviews | Thematic analysis using a deductive approach | No |

**^a^ 26 papers included duplicate studies amalgamated into 24 studies**

**^b^ 2 paper identified through other sources**
