# Supplementary material for: The Acceptability and Usability of Digital Health Interventions for Adults With Depression, Anxiety, and Somatoform Disorders: Qualitative Systematic Review and Meta-Synthesis
Source: J Med Internet Res. 2020 Jul 6;22(7):e16228. doi: 10.2196/16228 (PMC7381032; doi:10.2196/16228)
Supplement: Multimedia Appendix 7 [file jmir_v22i7e16228_app7.docx]

| First-order construct, second-order construct, and third-order construct: synthesis of findings | | | Subthemes |
| --- | --- | --- | --- |
| **Initial motivations and approaches to DHIs^a^** | | | |
| Positive: “My expectation was clearly to find a tool that would help stop the depression from reappearing in the future. Both finding a tool that will help me recognize when a depression is on its way, and a tool that can quickly be a way out of it so that I won't have to end up being as deeply depressed.” [1] | Participant expectation of a DHI was to find a tool to self-manage depression and prevent relapse | The prospect of using DHIs enabled a sense of empowerment through developing tools for self-management | Initial motivations: hope, accessibility, and cynicism |
| Negative: “I probably did come into it with lost heart, because I assumed from the start that I didn't think the computer programme was going to be for me, and maybe I sort of convinced myself.” [2] | Participant expectation of the DHI was that of skepticism | From the outset, the DHI was perceived not to be tailored to meet individual needs | Initial motivations: hope, accessibility, and cynicism |
| Positive: “You could sit there and just actually take your time to do it. You know you could really think. Whereas when you’re talking to somebody and you’ve got an hour or three-quarters of an hour or something, you really kind of you know...so time to me is the important thing.” [3] | The DHI enabled participants to have more time to work through and reflect on sessions | DHIs facilitated independent working and allowed more time for reflection and application | Participant approaches to engaging with a DHI: active versus passive |
| Negative: “At the end of the day, you still had to try and come up with the problems yourself and that’s quite difficult and I found it quite stressful . . . I mean, it’s hard doing it yourself, in isolation.” [4] | Participants found it difficult to identify issues on their own | The autonomous nature of DHIs can be perceived to be challenging and stressful | Participant approaches to engaging with a DHI: active versus passive |
| **Personalization of treatment** | | | |
| Positive: “Yeah. At the beginning I used to say I’ll do it every Wednesday morning, because Wednesday morning’s the best morning for me to do anything. I’ll go on it. But now I just go on it when I feel like it, and I find that better. I think that’s one of the bonuses of it, you can do it when you want to do it.” [5] | Flexibility and ease of access to therapy was perceived positively by some | The flexible and accessible nature of DHIs makes it easier for participants to choose when to complete the sessions | Flexibility and autonomy |
| Negative: “When you've got your off days it's easier to not bother with the computer whereas, you know, if you've got a face to face it's not, to me, I think it's not polite to not turn up. So I think yeah it's definitely going to work, you know, against it being so flexible.” [6] | A negative aspect of the flexibility of DHIs was that it was easier to avoid | The flexible and autonomous nature of DHIs made it easier for participants to choose not to complete sessions; there was no sense of obligation when participants were feeling less positive | Flexibility and autonomy |
| Positive: “I generally feel pretty confident and happy and would not label myself as somebody with anxiety or any of those issues so I don't think I would have accessed help in another way because I didn't really want to be labelled as you know as an anxious or depressed or whatever.” [7] | DHIs can be more accessible because it reduces the anxiety of being labelled with a mental health condition | DHIs were perceived to be more appealing because it reduced the stigma of accessing treatment for psychological problems | Stigma and privacy |
| Negative: “I think, probably face to face is necessary, I think, you have to actually get over that shame, if possible.” [5] | Some participants felt that face-to-face contact is important in overcoming shame | Face-to-face contact was perceived to be essential in overcoming the stigma | Stigma and privacy |
| Positive: “It was helpful to kind of see your progress and unlocking goals at the end of it. It was good to have a sense of progress through after completing each module.” [8] | Participants liked to be able to monitor progress after each module | Functionality of DHIs such as being able to monitor and reflect on progress was perceived positively | Functionality, content, and interface |
| Negative: “I thought that it was too much to read, and I cannot read anything at all that I need to remember or learn. It goes in here and out there [pointing at the ears].” [9] | The session content was too long, making it difficult to retain the information | Lengthy sessions had an impact on retaining focus | Functionality, content, and interface |
| **The value of receiving personal support in DHIs** | | | |
| Positive: “The book wasn’t completely useless, but if I’d have just had the book without the therapist I don’t think I would have made the improvements that I did do… when I talked about it with him, even thought it was basically common sense what he was telling me, because I’d never thought about it because that was my life the way it was, it was helpful the fact that he was putting everything into context for me.” [1] | Having a therapist to discuss sessions with in addition to the computerized therapy was helpful as it put things into context | Access to a therapist in addition to computerized sessions facilitated improvement and outcome | Support to understand DHIs |
| Negative: “I thought that the computer program was the questionnaire, and the doctor. So however I read it I thought computer program and doctor. That was it for me: questionnaire and doctor.” [10] | The research elements were misinterpreted to be the therapy | Without additional support, participants struggled to distinguish between the research and therapy | Support to understand DHIs |
| Positive: “I had a therapist who called me and checked how I had proceeded and pushed me a bit and said ‘come on, go through this chapter until tomorrow, I’ll call you back then’. I needed someone to push me because I had a problem with sitting down and getting things done, to pursue things.” [11] | Having a therapist encouraged motivation to continue with sessions for people who lacked motivation | Therapist contact encouraged motivation to complete tasks | Support to enhance commitment and motivation |
| Negative: “From my point of view, the contact with the therapist was an essential aspect of therapy. Therefore, I lost all my interest in the therapy and didn't want to continue.” [12] | Therapist contact was seen as vital and a lack of it influenced decisions to discontinue therapy | Lack of therapist contact impact discouraged participant willingness to continue with therapy | Support to enhance commitment and motivation |
| Negative: “I was feeling that the therapy wasn't going to help me with my problems. I thought it could lead me to be even more anxious and that it wasn't going to be beneficial for me. So, I felt that I was going to waste my time if I continued.” [12] | The decision to discontinue with the DHI was because continuing with sessions was perceived to increase anxiety symptoms | The DHI was perceived to potentially increase anxieties. This discouraged continuation with therapy. | Suitability and the desire for additional support |
| Positive: “I felt like I was just chatting away, that was the good thing, I was talking to someone who was listening to me...I was talking to a person. I wasn’t typing on a machine.” [13] | Participants were able to establish a good rapport with the therapist and expressed that it felt like a face-to-face interaction | Participants were able to develop a therapeutic relationship with the therapist via written communication. They felt listened to, and it did not feel mechanical. It paralleled a face-to-face session | Support to develop a virtual therapeutic relationship |
| Negative: “One disadvantage for the therapist's work is that his view to my body-language is limited. This must be communicated verbally and direct. I think in a face-to-face setting, more emotions can be transported […] this might make the therapist's work harder.” [14] | The disadvantage of videoconferencing was not being in the same room. This made it more challenging for the therapist to ascertain a participant’s expression of emotions | In DHIs, body language is limited compared with face-to-face interactions. The loss of body language and eye contact affected the therapeutic relationship | Support to develop a virtual therapeutic relationship |

^a^DHIs: digital health interventions.

**References**

1. Ly KH, Janni E, Wrede R, Sedem M, Donker T, Carlbring P, Andersson G. Experiences of a guided smartphone-based behavioral activation therapy for depression: a qualitative study. Internet Interv 2015 Mar; 2(1):60-8[ CrossRef ]
2. Gega L, Smith J, Reynolds S. Cognitive behaviour therapy (CBT) for depression by computer vs therapist: patient experiences and therapeutic processes. Psychother Res 2013; 23(2):218-31[ Medline ][ CrossRef ]
3. Donkin L, Glozier N. Motivators and motivations to persist with online psychological interventions: a qualitative study of treatment completers. J Med Internet Res 2012 Jun 22; 14(3):e91[ FREE Full text ][ Medline ][ CrossRef ]
4. Hind D, O'Cathain A, Cooper CL, Parry GD, Isaac CL, Rose A, Martin L, Sharrack B. The acceptability of computerised cognitive behavioural therapy for the treatment of depression in people with chronic physical disease: a qualitative study of people with multiple sclerosis. Psychol Health 2010 Jul; 25(6):699-712[ Medline] [CrossRef]
5. Knopp-Hoffer J, Knowles S, Bower P, Lovell K, Bee PE. 'One man's medicine is another man's poison': a qualitative study of user perspectives on low intensity interventions for obsessive-compulsive disorder (OCD). BMC Health Serv Res 2016 May 18; 16:188[ FREE Full text ][ Medline ][ CrossRef ]
6. Knowles SE, Lovell K, Bower P, Gilbody S, Littlewood E, Lester H. Patient experience of computerised therapy for depression in primary care. BMJ Open 2015 Nov 30; 5(11):e008581[ FREE Full text ][ Medline ][ CrossRef ]
7. Ashford MT, Olander EK, Rowe H, Fisher JR, Ayers S. Feasibility and acceptability of a web-based treatment with telephone support for postpartum women with anxiety: randomized controlled trial. JMIR Ment Health 2018 Apr 20; 5(2):e19[ FREE Full text ][ Medline ][ CrossRef ]
8. Walsh A, Richards D. Experiences and engagement with the design features and strategies of an internet-delivered treatment programme for generalised anxiety disorder: a service-based evaluation. Br J Guid Counc 2016 Feb 28; 45(1):16-31[ CrossRef ]
9. Johansson O, Michel T, Andersson G, Paxling B. Experiences of non-adherence to internet-delivered cognitive behavior therapy: a qualitative study. Internet Interv 2015 May; 2(2):137-42[ CrossRef ]
10. Gerhards SA, Abma TA, Arntz A, de Graaf LE, Evers SM, Huibers MJ, Widdershoven GA. Improving adherence and effectiveness of computerised cognitive behavioural therapy without support for depression: a qualitative study on patient experiences. J Affect Disord 2011 Mar; 129(1-3):117-25[ Medline ][ CrossRef ]
11. Holst A, Nejati S, Björkelund C, Eriksson MC, Hange D, Kivi M, Wikberg C, Petersson E. Patients' experiences of a computerised self-help program for treating depression - a qualitative study of internet mediated cognitive behavioural therapy in primary care. Scand J Prim Health Care 2017 Mar; 35(1):46-53[ FREE Full text ][ Medline ][ CrossRef ]
12. Fernández-Álvarez J, Díaz-García A, González-Robles A, Baños R, García-Palacios A, Botella C. Dropping out of a transdiagnostic online intervention: a qualitative analysis of client's experiences. Internet Interv 2017 Dec; 10:29-38[ FREE Full text ][ Medline ][ CrossRef ]
13. Beattie A, Shaw A, Kaur S, Kessler D. Primary-care patients' expectations and experiences of online cognitive behavioural therapy for depression: a qualitative study. Health Expect 2009 Mar; 12(1):45-59[ FREE Full text ][ Medline ][ CrossRef ]
14. Etzelmueller A, Radkovsky A, Hannig W, Berking M, Ebert DD. Patient's experience with blended video- and internet based cognitive behavioural therapy service in routine care. Internet Interv 2018 Jun; 12:165-75[ FREE Full text ][ Medline ][ CrossRef ]
